# A neural signature for gastrointestinal symptoms in depression: insula-gastric connectivity predicts symptom severity

**DOI:** 10.3389/fpsyt.2025.1672148

**Published:** 2025-11-19

**Authors:** Li Qi, Ting Zhang, Xiaomin Pan, Zhishun Gao, Jin Li, Yue Yu, Wenjia Wang, Qiang Wei, Jin-Ying Yang, Kai Wang, Tongjian Bai, Qianqian Li

**Affiliations:** 1 Department of Neurology, The First Affiliated Hospital of Anhui Medical University, Hefei, China; 2 Department of Psychology and Sleep Medicine, The Second Affiliated Hospital of Anhui Medical University, Hefei, China; 3 Department of Psychiatry, The First Affiliated Hospital of Anhui Medical University, Hefei, China; 4 Department of Neurology, The Second Affiliated Hospital of Anhui Medical University, Hefei, China; 5 Laboratory Center for Information Science, University of Science and Technology of China, Hefei, China; 6 Medical Imaging Center, Department of Electronic Engineering and Information Science, University of Science and Technology of China, Hefei, China; 7 The School of Mental Health and Psychological Sciences, Anhui Medical University, Hefei, China; 8 Institute of Artificial Intelligence, Hefei Comprehensive National Science Center, Hefei, China; 9 Anhui Province Key Laboratory of Cognition and Neuropsychiatric Disorders, Hefei, China; 10 Collaborative Innovation Center of Neuropsychiatric Disorders and Mental Health, Hefei, China; 11 Anhui Institute of Translational Medicine, Hefei, China

**Keywords:** major depressive disorder, gastrointestinal symptoms, insula, gastric network, biomarker

## Abstract

**Background:**

Gastrointestinal (GI) symptoms are a common and burdensome dimension of major depressive disorder (MDD), yet their neurobiological underpinnings are poorly understood. It is unclear how the brain’s processing of visceral signals relates to the subjective experience of GI distress in depression. We aimed to identify a neural substrate for GI symptoms by examining functional connectivity (FC) between the insula and a network defined by gastric rhythms.

**Methods:**

We first identified a gastric-related seed in the posterior insula (GD-pINS) using a large normative dataset of 652 healthy adults. Subsequently, 100 MDD patients—stratified into groups with (GD; n=58) and without (NGD; n=42) GI symptoms—and 80 healthy controls (HCs) were recruited. Using resting-state fMRI, we analyzed FC between the GD-pINS and the gastric network (GN). Group differences, clinical correlations, and the utility of FC features for patient classification via a support vector machine (SVM) were assessed.

**Results:**

Compared to HCs, MDD patients as a whole showed reduced GD-pINS to GN connectivity. Paradoxically, GD patients exhibited relatively stronger connectivity than NGD patients. This symptom-specific enhancement was driven by pathways connecting the posterior insula to the secondary somatosensory cortex (SII). The strength of this insula-SII connection was positively correlated with GI symptom severity. An SVM classifier using these connectivity features distinguished between GD and NGD patients with high accuracy (AUC = 0.82).

**Conclusions:**

Our findings reveal a distinct neural signature for GI distress in depression, characterized by aberrant connectivity within an insula-somatosensory circuit. This circuit, which shows relative enhancement in symptomatic patients against a backdrop of globally reduced connectivity, may reflect a mechanism of somatosensory amplification. It represents a potential biomarker for patient stratification and a novel target for therapeutic intervention.

## 1 Introduction

Major Depressive Disorder (MDD) is a psychiatric condition characterized by high prevalence, recurrence, and disability rates (1–4), posing a significant global public health challenge (5). The clinical presentation of MDD is not confined to core affective symptoms such as low mood and anhedonia but is frequently accompanied by a complex array of somatic symptoms, including pain and fatigue (6). Approximately two-thirds of patients with depression initially present at primary care settings with complaints of physical discomfort, and these somatic symptoms often mask the underlying emotional distress (7).

Gastrointestinal (GI) symptoms are among the most prevalent and distressing manifestations for patients with depression. These symptoms include abdominal pain, nausea, constipation, or bloating (8). The presence of these symptoms predicts a more severe disease course and a higher risk of relapse (9, 10). They also present a formidable diagnostic challenge. This can lead to ineffective medical investigations, which exacerbates patient suffering and the burden on healthcare systems (11, 12). This highlights the limitations of current diagnostic paradigms, which rely heavily on subjective symptom reporting (13, 14). Consequently, the development of objective biomarkers that link the physical discomfort of GI symptoms to underlying neural circuit dysfunction has emerged as a critical research imperative in psychiatry (15, 16).

A growing body of evidence indicates that MDD is closely associated with significant dysfunction in interoception (17)—the perception of the body’s internal physiological state. One form of this abnormality involves hypervigilance and a negative cognitive bias toward normal, innocuous physiological sensations, a phenomenon known as somatosensory amplification (18). Within the complex neural circuitry responsible for interoceptive processing, the insular cortex (insula) plays a pivotal role as a central hub (19–21). The insula receives ascending visceral information from the brainstem (e.g., the nucleus of the solitary tract). It then integrates this information with higher-order psychological processes such as emotion, cognition, and motivation. This integration ultimately forms a coherent, subjective awareness of the body’s internal state (22). Insular activation is correlated with the subsequently experienced intensity of touch or pain (23–25), directly linking subjective pain perception with insular cortical function. A substantial body of neuroimaging research consistently demonstrates that the insula in patients with depression exhibits significant structural and functional abnormalities. These abnormalities are closely related to their somatic presentations (22, 26). Furthermore, alterations in the insula’s functional connectivity (FC) patterns are associated with somatic symptoms. Increased connectivity between the insula and the frontoparietal lobe, fusiform gyrus, and cerebellum has been linked to more severe somatic symptoms or related conditions, such as pain in fibromyalgia (27–33), underscoring the central role of insular activation in somatic symptomatology.

The insula is not a functionally homogenous structure; it exhibits significant functional heterogeneity (34, 35). A global analysis of the insula fails to capture the specific insular activity most relevant to gastrointestinal function. Therefore, identifying a gastric-centric hub within the insula is a critical step.

The bidirectional communication along the gut-brain axis serves as the physiological foundation of interoception (36, 37). Concurrently, the study of resting-state networks (RSNs) provides a powerful avenue for exploring the brain’s functional architecture in both health and disease (38). The seminal work of Rebollo et al. (2018) identified a novel, rhythm-based RSN termed the gastric network (GN) (39). This was the first study to reveal a direct, dynamic coupling between the intrinsic rhythm of the stomach and core brain regions responsible for bodily representation. This finding has since been corroborated by other researchers (40). Further animal studies have demonstrated that vagotomy can disrupt this stomach-brain coupling (41), while transcutaneous auricular vagus nerve stimulation (taVNS) can modulate it (42). This series of findings underscores the reliability and broad application potential of the GN.

However, despite numerous studies linking insular activation to somatic symptoms, no research to date has connected the activity of the GN with the subjective experience of gastrointestinal somatic symptoms in MDD. This represents a critical gap in understanding the neurobiology of somatic depression. To bridge this gap, we formulated a central hypothesis: that a specific locus within the insula, one exhibiting the strongest functional coupling with gastric-related brain activity, serves as a key neural substrate for GI distress in depression. We posited that the FC between this insular locus and the broader GN would exhibit symptom-specific alterations in MDD patients with prominent GI symptoms compared to those without. Furthermore, we hypothesized that the strength of these specific neural pathways would be directly correlated with the severity of patients’ reported GI symptoms and could therefore serve as a potential neuroimaging biomarker for stratifying patient subgroups. To systematically test this hypothesis, our study was designed to achieve three primary objectives: First, to precisely identify and localize a functionally-defined seed point within the insula that is most robustly coupled with the GN. To achieve this, we utilized a large-scale normative human connectome dataset, ensuring the identified region is representative of a general healthy population. Second, to investigate differences in FC between this insular seed and the GN by comparing three distinct groups: MDD patients with notable GI symptoms, MDD patients without such symptoms, and a matched group of healthy controls. This comparative analysis was designed to assess connectivity with both the entire GN and its finer-grained constituent regions, allowing us to pinpoint specific pathways underlying symptom presentation. Finally, to evaluate the clinical utility of these connectivity findings by constructing a machine learning classification model. This final step aimed to determine whether the identified FC features possess sufficient predictive power to serve as an objective biomarker for distinguishing between the MDD patient subgroups based on their neural signatures.

## 2 Materials and methods

### 2.1 Study context

**Figure 1:**
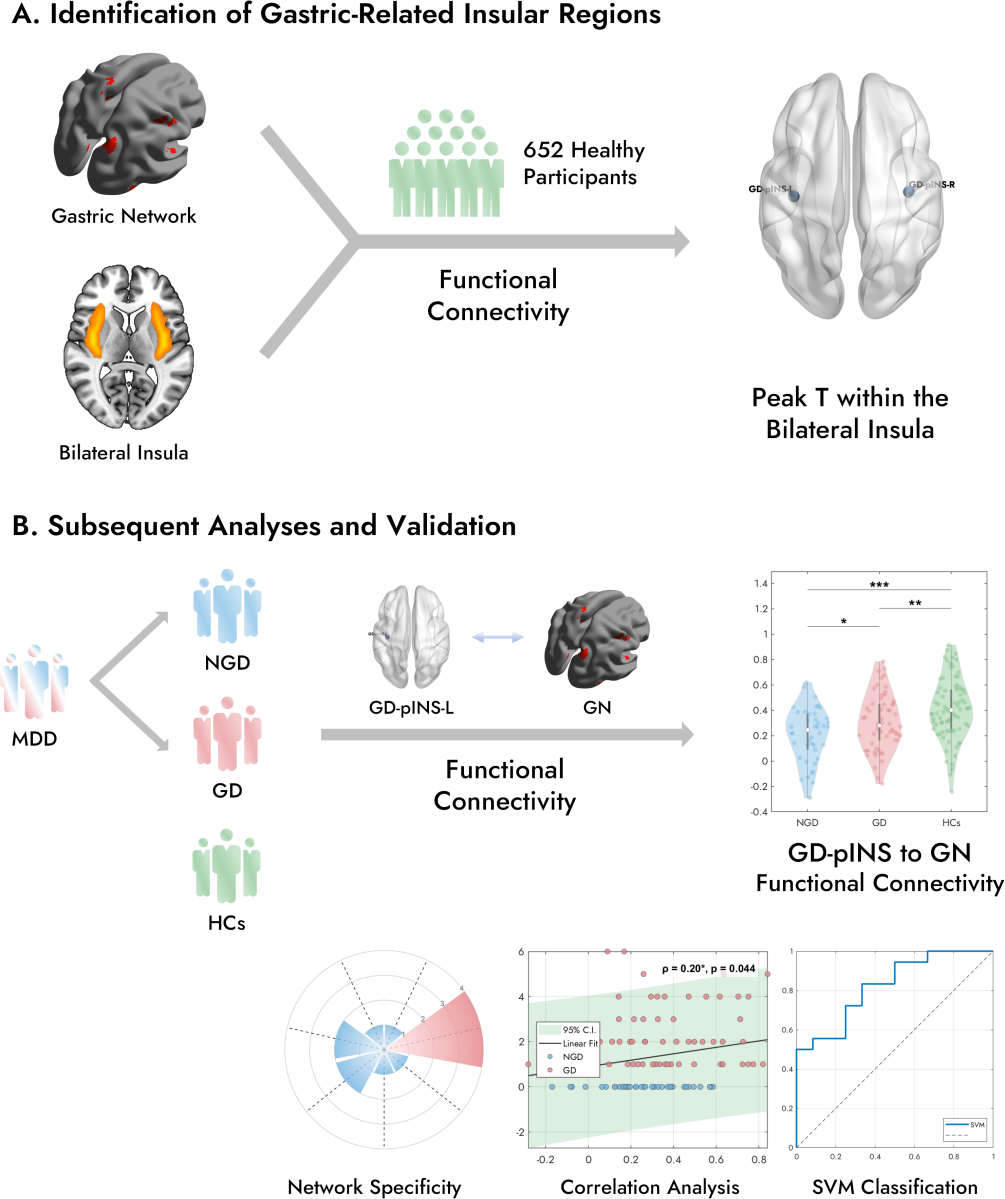
Overview of this study. **(A)** In a large cohort of 652 healthy participants, FC analysis was performed between the bilateral insula and a previously established gastric network. This analysis identified the peak T-value within the posterior portion of the bilateral insula that exhibited the strongest FC with the GN, defining the GD-pINS. **(B)** Three groups were included in the subsequent analyses: patients with MDD with gastrointestinal symptoms (GD), MDD patients without GI symptoms (NGD), and healthy controls (HCs). FC was examined between the left GD-pINS and the GN across the three groups. The violin plot illustrates the distribution of GD-pINS-GN FC in each group, with significant difference. Further analyses included network specificity evaluation and machine learning-based classification. MDD, Major Depressive Disorder; FC, Functional Connectivity; GD-pINS, Gastric-Defined Posterior Insula. *p < 0.05, **p < 0.01, ***p < 0.001.

An overview of the study design is presented in **Figure 1**. This study aimed to elucidate the neural underpinnings of GI symptoms in MDD by integrating findings from a large normative imaging dataset with those from a clinical cohort. Our primary objectives were twofold: First (**Figure 1A**), to identify a specific insular region functionally coupled with a predefined gastric-related brain network using the normative data. Second (**Figure 1B**), to investigate how functional connectivity between this identified insular region and the gastric network differs among MDD patients with and without GI symptoms and healthy controls, and whether such connectivity patterns relate to symptom severity and could potentially differentiate patient subgroups. The subsequent analyses involved assessing group differences in functional connectivity, clinical correlations, and classification performance based on these neural features.

### 2.2 Participants

The gastric-related insular regions of interest (ROIs) were identified using resting-state functional MRI (fMRI) data from a large-scale normative connectome dataset comprising 652 healthy adults. Participants were recruited through public advertisements, and data collection was conducted at the University of Science and Technology of China in Hefei, China. The imaging parameters were consistent with those used in the main study cohort. Detailed information regarding the data acquisition for this cohort has been described in our previous publications (43, 44).

For the main study, a cohort of 100 patients was recruited from the Emotional Disorder Clinic at the Anhui Mental Health Center in Hefei, China. Each patient received a primary diagnosis of MDD, established according to the Diagnostic and Statistical Manual of Mental Disorders, Fifth Edition (DSM-5). This diagnosis was independently confirmed by two licensed psychiatrists using structured clinical interviews. Inclusion criteria for patients were: (1) a current diagnosis of MDD; (2) being in a depressive state at the time of scanning; (3) right-handedness; and (4) an age range of 18 to 65 years. Exclusion criteria for all participants encompassed: (1) any history of neurological disorders; (2) comorbid psychiatric conditions such as substance use disorder, schizophrenia, or bipolar disorder; (3) contraindications to MRI scanning, including metal implants; and (4) excessive head motion during imaging (defined as translation > 3 mm or rotation > 3°). Concurrently, a group of 80 Healthy Controls (HCs) was recruited via local advertisements. These individuals were free from any reported emotional or somatic symptoms and other health issues and were matched to the patient group on age, sex, and education. The same exclusion criteria applied to the patient group were also used for the HCs.

### 2.3 Assessment of clinical symptoms and subgrouping

Gastrointestinal symptoms were quantified using a composite score derived from the Patient Health Questionnaire-15 (PHQ-15) (45), a widely used and internationally validated instrument for assessing somatic symptom burden (46, 47). Specifically, we created a Gastrointestinal Discomfort Index (GDI) by summing the scores of three items related to gastrointestinal distress: stomach pain, constipation/diarrhea, and nausea/gas/indigestion (see **Supplementary Table 1** for details). These specific items were selected from the PHQ-15 as they are the most direct measures of core GI dysfunction, allowing for a targeted assessment of this symptom cluster distinct from the more general somatic complaints in the full scale (48, 49). Each item is rated on a 3-point scale (0–2), yielding a total GDI score ranging from 0 to 6. Based on this index, MDD patients were stratified into two subgroups: those with a GDI score of 0 were assigned to the Depression without gastrointestinal symptoms (NGD) group, while those with a score of 1 or higher were assigned to the Gastrointestinal Depression (GD) group.

### 2.4 Neuroimaging data acquisition

All MRI data were collected on a 3.0T GE Discovery MR750 scanner at the University of Science and Technology of China. For the duration of the scan, participants were instructed to lie still with their eyes closed, stay awake, and avoid systematic head movements. A high-resolution T1-weighted anatomical scan was acquired first using a sagittal sequence with the following parameters: TR = 8.16 ms, TE = 3.18 ms, flip angle = 12°, slice thickness = 1 mm, FOV = 256 × 256 mm², and voxel size = 1 × 1 × 1 mm³. Subsequently, resting-state functional images were obtained with an echo-planar imaging (EPI) sequence (TR = 2400 ms, TE = 30 ms, flip angle = 90°, 46 slices, slice thickness = 3 mm, FOV = 192 × 192 mm², matrix = 64 × 64, voxel size = 3 × 3 × 3 mm³). Each functional run lasted 8 minutes and 41 seconds, yielding 217 volumes.

### 2.5 Neuroimaging data preprocessing

Resting-state fMRI data were preprocessed using the Resting-State fMRI Data Analysis Toolkit plus (RESTplus, v1.28; http://www.restfmri.net) (50), which operates within the Statistical Parametric Mapping (SPM12; www.fil.ion.ucl.ac.uk/spm) (51) framework. The initial 10 volumes from each participant’s scan were discarded to allow for T1 signal stabilization. The remaining volumes underwent slice-timing correction and were then realigned to the first volume to correct for head motion. Any participant exhibiting head motion greater than 3 mm in translation or 3° in rotation was excluded from the analysis. Individual T1-weighted images were co-registered to the functional data and subsequently normalized to Montreal Neurological Institute (MNI) space via the Diffeomorphic Anatomical Registration Through Exponentiated Lie Algebra (DARTEL) toolbox. The resulting transformation parameters were applied to the functional images. Finally, the normalized functional data were smoothed with a 6-mm full-width at half-maximum (FWHM) Gaussian kernel, detrended, and band-pass filtered (0.01–0.08 Hz). To minimize the influence of non-neuronal noise, nuisance covariates—including the six head motion parameters, the mean white matter signal, and the mean cerebrospinal fluid signal—were regressed out. In line with current recommendations, global signal regression was not applied to avoid spurious negative correlations and the loss of neuronally relevant variance (52, 53).

### 2.6 Identification of the gastric-related insula regions

To precisely locate the insular subregions most robustly connected to the GN, we first utilized the GN mask reported by Rebollo et al. (2018) (39). This network was originally defined based on significant phase synchrony between the electrogastrogram (EGG) and BOLD signals in healthy adults, and the mask is publicly available (NeuroVault ID: 51888). This network encompasses cortical and subcortical areas such as the postcentral gyrus, superior temporal gyrus, supplementary motor area, cingulate gyrus, and precuneus. We then performed a voxel-wise FC analysis within an bilateral insula mask. Specifically, for each participant in the large normative dataset (n=652), we computed the Pearson correlation between the mean time series of the GN and the time series of every voxel within the insula. The resulting individual correlation maps were transformed to z-scores and entered into a one-sample t-test. This group-level analysis produced a statistical map highlighting the connectivity pattern of the gastric-insula system. The peak voxels with the highest positive T-values in the left and right insula were identified. Subsequently, these peak coordinates served as the centers for two 6-mm radius spherical ROIs, which constituted the final seeds for subsequent analyses.

### 2.7 FC analysis

Following preprocessing, two primary sets of FC analyses were conducted. First, whole-network FC was computed by correlating the mean time series of the left and right gastric-related insula regions with the mean time series of the entire GN mask. Second, seed-to-seed FC was calculated between the bilateral gastric-related insula regions and 39 individual nodes within the GN. These nodes were defined as 6-mm radius spheres centered on the peak coordinates reported by Rebollo et al. (2018; see **Supplementary Table 2**) (39). All resulting correlation coefficients were Fisher’s z-transformed.

### 2.8 Statistical analysis

All statistical analyses were performed using MATLAB R2024b (The MathWorks, Inc.) and SPSS version 26.0 (SPSS Inc., Chicago, IL, USA). Demographic and clinical variables were compared using One-way Analysis of Variance (ANOVA) for continuous variables across the three groups, with Welch’s t-tests for two-group comparisons. The homogeneity of variances was tested prior to each ANOVA. Categorical variables were assessed using the chi-square (χ²) test. To investigate the clinical relevance of altered connectivity and assess potential confounds, correlation analyses were conducted between the FC values of significant connections and both GDI scores and illness duration across all patients. Finally, to characterize the network-level properties of the findings, the significantly altered connections were categorized into the functional subnetworks defined by Rebollo et al. (2018) (39) to calculate their distribution. For all tests, an uncorrected threshold of p < 0.05 was considered statistically significant.

### 2.9 Machine learning classification

To provide a preliminary evaluation of the potential of the identified neuroimaging features as biomarkers, we developed a support vector machine (SVM) classifier to distinguish between NGD and GD patients. The analysis was implemented in MATLAB using the LIBSVM toolbox (54). The feature set comprised a combination of demographic data (age, sex, education) and FC measures. The patient data were randomly partitioned into a training set (70%) and a held-out test set (30%). To prevent data leakage, feature normalization parameters (scaling to [-1, 1]) were learned from the training set only and then applied to both the training and test sets. We employed an SVM with a radial basis function (RBF) kernel. A grid-search strategy combined with 5-fold cross-validation was conducted on the training set to identify the optimal hyperparameters for C (cost) and γ (gamma). The model was then retrained on the entire training set using these optimal parameters. Finally, the classifier’s performance was evaluated on the independent test set.

## 3 Results

### 3.1 Demographic and clinical characteristics

The demographic and clinical data are summarized in **Table 1**. Within the patient cohort, based on their GDI scores, 42 individuals were classified into the NGD group and 58 into the GD group. There were no significant differences among the three groups (NGD, GD, and HCs) in terms of age (F = 0.722, p = 0.487), gender (χ² = 1.662, p = 0.436), or years of education (F = 1.660, p = 0.193). Patients in the GD group reported significantly higher scores on both the PHQ-15 (T = -7.686, p < 0.001) and the GDI (T = -10.286, p < 0.001) compared to the NGD group. Interestingly, the NGD group had a significantly longer duration of illness than the GD group (T = 2.853, p = 0.005). However, illness duration did not significantly correlate with the GDI-associated FC values (p > 0.05). The two patient subgroups did not differ significantly in the overall rate of medication use or in the prescription rates for specific medication classes, such as SSRIs and SNRIs.

**Table 1:** Comparison of demographic and clinical characteristics across study groups.

Characteristics	NGD (n = 42)	GD (n = 58)	HCs (n = 80)	Statistic (F/T/χ²)	*p* value
Age (years)	40.95 (12.45)	39.47 (13.62)	42.54 (16.80)	0.722	0.487
Gender (male/female)	15/27	14/44	25/55	1.662	0.436
Education (years)	10.33 (4.35)	8.98 (4.70)	10.19 (4.03)	1.660	0.193
PHQ-15	5.88 (2.91)	11.21 (4.20)		-7.686^***^	<0.001
GDI	0.00 (0.00)	2.34 (1.42)		-10.286^***^	<0.001
Duration of illness (months)	108.38 (100.83)	57.74 (76.72)		2.853^**^	0.005
Medication (patients, n)	41	56		0.095	0.756
SSRIs	22	34		0.385	0.54
SNRIs	16	22		0.000	0.987
antipsychotics	25	31		0.365	0.546
BZDs	2	6		1.032	0.310
NBZDs	9	18		1.140	0.286

Values are presented as mean (standard deviation) for continuous variables and count (n) for categorical variables. P-values for three-group comparisons (Age, Education) were obtained from one-way analysis of variance (ANOVA). P-values for two-group comparisons between NGD and GD were obtained from Welch’s t-test for continuous variables and chi-square tests (χ²) for categorical variables. NGD, Depression without gastrointestinal symptoms; GD, Gastrointestinal Depression; HCs, Healthy Controls; PHQ-15, Patient Health Questionnaire-15; GDI, Gastrointestinal Discomfort Index; SSRIs, selective serotonin reuptake inhibitors; SNRIs, serotonin-norepinephrine reuptake inhibitors; BZDs, benzodiazepines; NBZDs, non-benzodiazepines. **p < 0.01, ***p < 0.001.

### 3.2 Location of the bilateral GD-pINS

**Figure 2:**
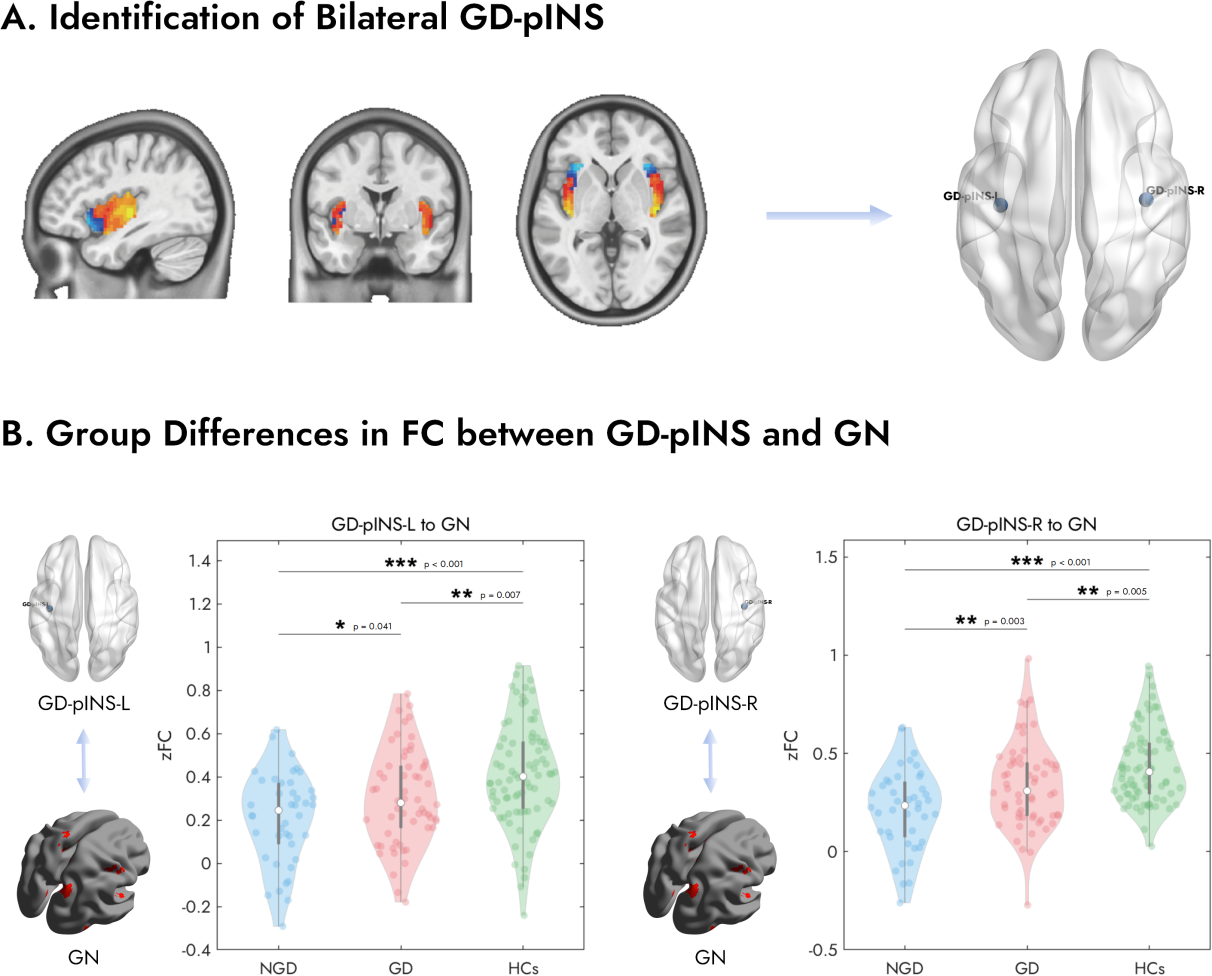
Identification of the bilateral GD-pINS and its altered FC with the gastric network across study groups. **(A)** The GD-pINS ROIs were identified from a large normative cohort of 652 healthy participants. Brain maps display regions within the bilateral insula where FC with the GN was positively (hot colors) or negatively (cool colors) correlated. The peak voxels exhibiting the strongest positive correlation in the left and right posterior insula were defined as the centers for the GD-pINS-L and GD-pINS-R ROIs, respectively. **(B)** Violin plots illustrate the FC (Fisher’s z-transformed correlation, zFC) between the left/right GD-pINS and the entire GN for three groups: NGD, GD, and HCs. Both patient groups showed significantly reduced FC compared to HCs. Critically, the GD group exhibited significantly higher FC than the NGD group for both the left and right GD-pINS. GD-pINS, Gastric-Defined Posterior Insula; ROIs, regions of interest; FC, functional connectivity; NGD, Depression without gastrointestinal symptoms; GD, Gastrointestinal Depression; HCs, Healthy Controls. *p < 0.05, **p < 0.01, ***p < 0.001.

As illustrated in **Figure 2A**, the analysis in the normative cohort identified two peak voxels of maximal connectivity with the GN, located symmetrically in the posterior insula of each hemisphere. The MNI coordinates for these peaks were (39, -15, 0) for the right hemisphere and (-39, -18, 0) for the left hemisphere. Given their anatomical location, these regions were termed the Gastric-Defined Posterior Insula (GD-pINS).

### 3.3 Altered FC between GD-pINS and the GN

We first examined the overall FC between the bilateral GD-pINS and the entire GN across the three groups (**Figure 2B**). A one-way ANOVA revealed a significant main effect of group for both the left GD-pINS (F = 11.83, p < 0.001) and the right GD-pINS (F = 16.45, p < 0.001). *Post-hoc* analyses on the left GD-pINS-GN connectivity showed that both the NGD (p < 0.001) and GD (p = 0.007) groups had significantly lower FC compared to HCs. Critically, the NGD group exhibited significantly lower FC than the GD group (p = 0.041). For the right GD-pINS-GN connectivity, a similar pattern emerged: both NGD (p < 0.001) and GD (p = 0.005) groups showed reduced FC relative to HCs. More importantly, the NGD group displayed significantly weaker FC than the GD group (p = 0.003).

### 3.4 Specific altered FC in MDD patients with GI symptoms

**Figure 3:**
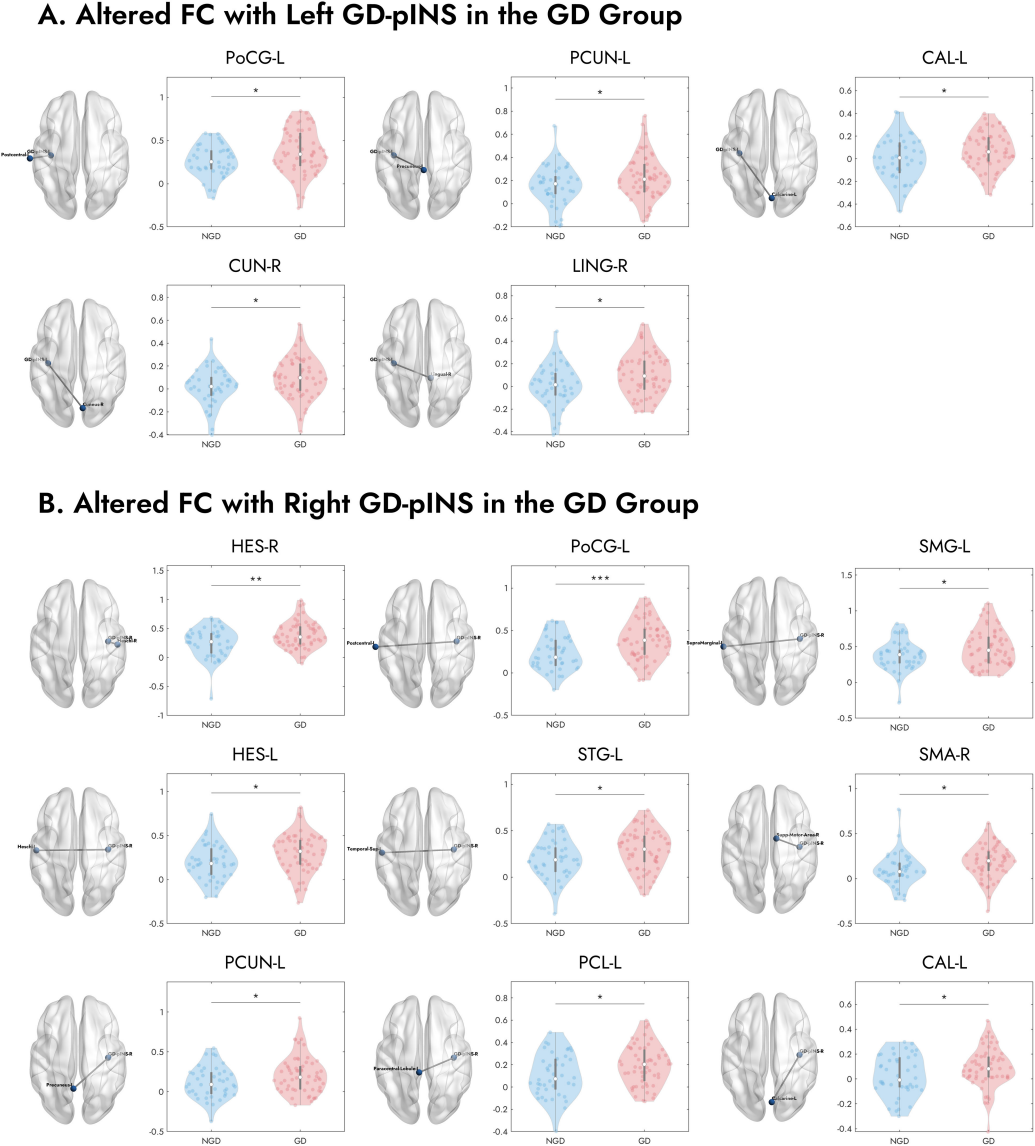
Visualization of specific brain regions with significantly increased FC to the bilateral GD-pINS in GD compared to NGD. **(A)** Brain regions exhibiting increased FC with the left GD-pINS (GD-pINS-L) in the GD group, including the left postcentral gyrus (PoCG-L), left precuneus (PCUN-L), left calcarine sulcus (CAL-L), right cuneus (CUN-R), and right lingual gyrus (LING-R). **(B)** Brain regions exhibiting increased FC with the right GD-pINS (GD-pINS-R) in the GD group. These primarily involve bilateral auditory and somatosensory cortices, supplementary motor area, and visual processing areas. NGD, Depression without gastrointestinal symptoms; GD, Gastrointestinal Depression; GD-pINS, Gastric-defined posterior insula; FC, functional connectivity. *p < 0.05, **p < 0.01, ***p < 0.001.

To pinpoint the specific pathways underlying the whole differences, we compared the seed-to-seed FC between the GD and NGD groups. As detailed in **Table 2** and visualized in **Figure 3**, the GD group, compared to the NGD group, showed significantly increased FC between the GD-pINS and several nodes within the GN. Specifically, for the left GD-pINS, increased FC was observed with five regions, including the left postcentral gyrus, left precuneus, left calcarine sulcus, right cuneus, and right lingual gyrus. For the right GD-pINS, increased FC was found with nine regions, most prominently involving the bilateral somatosensory cortices (left postcentral gyrus, left supramarginal gyrus), auditory cortices (bilateral Heschl’s gyrus, left superior temporal gyrus), the right supplementary motor area, and visual-parietal areas (left precuneus, left paracentral lobule, left calcarine sulcus).

**Table 2:** Brain regions with altered FC to bilateral GD-pINS.

Brain Regions	Abbreviation	MNI coordinate			T value	*p* value	Cohen’s *d*
		x	y	z			
Altered connectivity with Left GD-pINS							
Left Postcentral Gyrus	PoCG-L	-66	-22	22	2.435^*^	0.017	0.480
Left Precuneus	PCUN-L	0	-37	55	1.988^*^	0.049	0.401
Left Calcarine Sulcus	CAL-L	3	-76	16	2.155^*^	0.034	0.442
Right Cuneus	CUN-R	6	-76	28	2.490^*^	0.016	0.501
Right Lingual Gyrus	LING-R	9	-37	-2	2.617^*^	0.010	0.529
Altered connectivity with Right GD-pINS							
Right Heschl’s Gyrus	HES-R	51	-19	10	2.713**	0.008	0.555
Left Postcentral Gyrus	PoCG-L	-66	-22	22	3.441***	<0.001	0.689
Left Supramarginal Gyrus	SMG-L	-60	-25	16	2.155*	0.034	0.430
Left Heschl’s Gyrus	HES-L	-54	-16	7	2.311*	0.023	0.465
Left Superior Temporal Gyrus	STG-L	-54	-19	7	2.335*	0.022	0.472
Right Supplementary Motor Area	SMA-R	9	-4	52	2.380*	0.019	0.482
Left Precuneus	PCUN-L	-6	-55	73	2.154*	0.034	0.431
Left Paracentral Lobule	PCL-L	-6	-34	76	2.300*	0.024	0.469
Left Calcarine Sulcus	CAL-L	3	-76	16	2.020*	0.046	0.411

The table lists brain regions showing significantly lower FC in the NGD group compared to the GD group. The T values and p-values are derived from Welch’s t-tests. The MNI coordinates represent the peak voxel of each ROI in gastric network. NGD, Depression without gastrointestinal symptoms; GD, Gastrointestinal Depression; GD-pINS, Gastric-defined posterior insula; MNI, Montreal Neurological Institute; FC, functional connectivity. *p < 0.05, **p < 0.01, ***p < 0.001.

### 3.5 Correlation between FC and GDI scores

**Figure 4:**
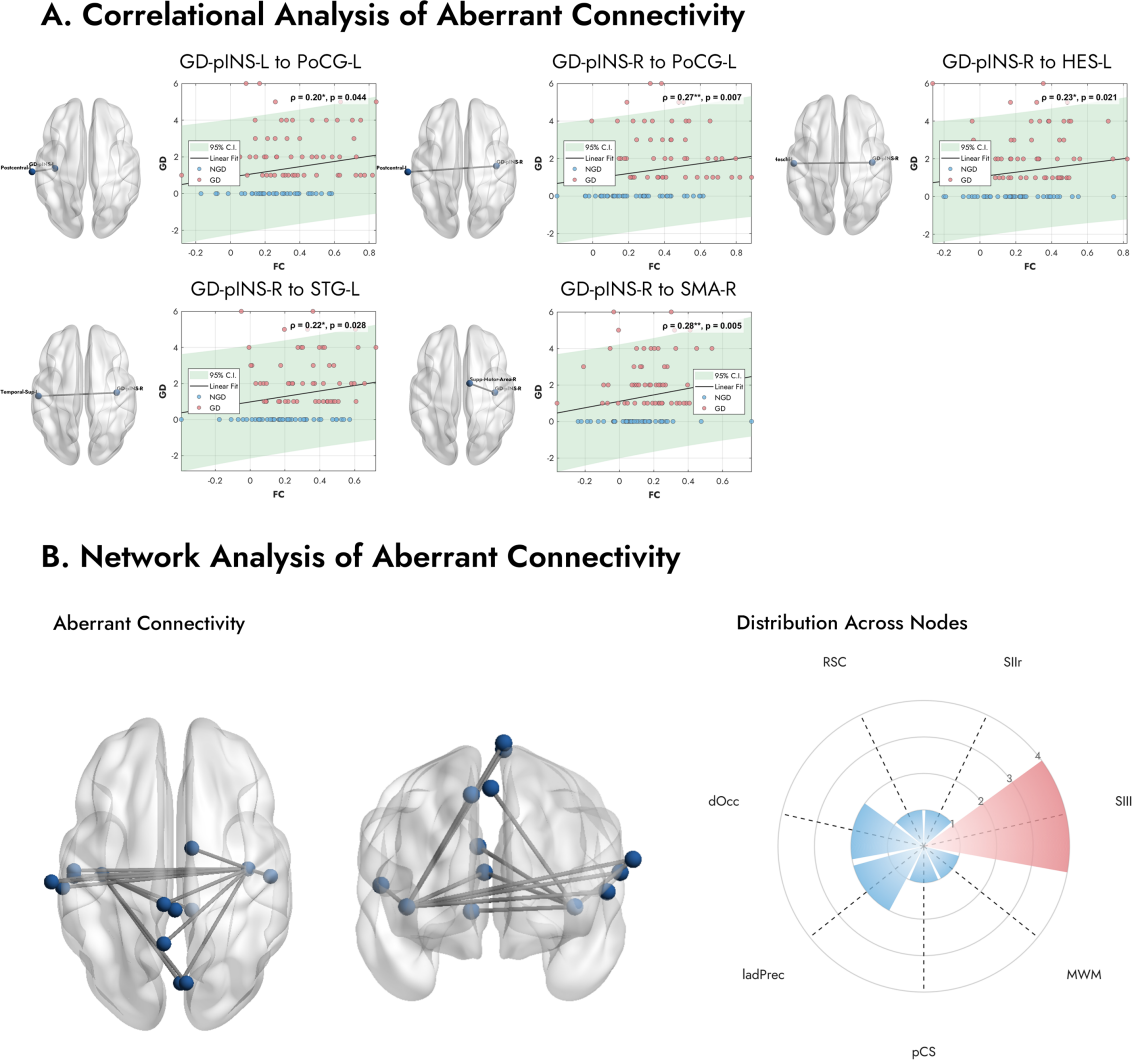
Correlation analysis and network properties of the aberrant functional connections. **(A)** Scatter plots display the relationship between FC of specific pathways and the GDI scores across all MDD patients. Stronger connectivity is associated with more severe gastrointestinal symptoms. **(B)** The number of abnormal connections in each network. The radar plot shows the distribution of these aberrant connections across the predefined functional networks from Rebollo et al. (2018) (39). A clear predominance is observed in the Secondary Somatosensory Left (SIIl) node, which contains the highest number of aberrant connections. *Note.* FC, Functional Connectivity; GDI, Gastrointestinal Discomfort Index. Abbreviations for networks are listed in **Supplementary Table 2**.

To assess the clinical relevance of these altered pathways, we examined the relationship between their FC values and GDI scores across all MDD patients (n=100). As depicted in **Figure 4A**, we found significant positive correlations, indicating that stronger connectivity in these specific pathways was associated with greater gastrointestinal symptom severity. Specifically, the FC between the right GD-pINS and the right supplementary motor area (ρ = 0.28, p = 0.005), the left postcentral gyrus (ρ = 0.27, p = 0.007), the left Heschl’s gyrus (ρ = 0.23, p = 0.021), and the left superior temporal gyrus (ρ = 0.22, p = 0.028) were all positively correlated with GDI scores. Additionally, the connectivity between the left GD-pINS and the left postcentral gyrus also showed a significant positive correlation (ρ = 0.20, p = 0.044).

### 3.6 Network distribution of aberrant connectivity

The left panel of **Figure 4B** illustrates all 14 connections exhibiting significantly altered FC in the GD group. To explore the spatial distribution of these connections, we mapped the associated brain regions onto the predefined GN nodes described by Rebollo et al. (2018) (39). As shown in the right panel of **Figure 4B**, a notable finding was the marked predominance of connections involving the Secondary Somatosensory Left (SIIl) node. Among the 12 aberrant regions identified, four (33.3%) belonged to the SII node. Other frequently involved networks included the Dorsal Occipital (dOcc) and the Left Anterior Dorsal Precuneus (ladPrec) nodes. These results suggest that functional disruptions in MDD patients with gastrointestinal symptoms are particularly concentrated within somatosensory processing pathways.

### 3.7 Performance of the SVM classifier

**Figure 5:**
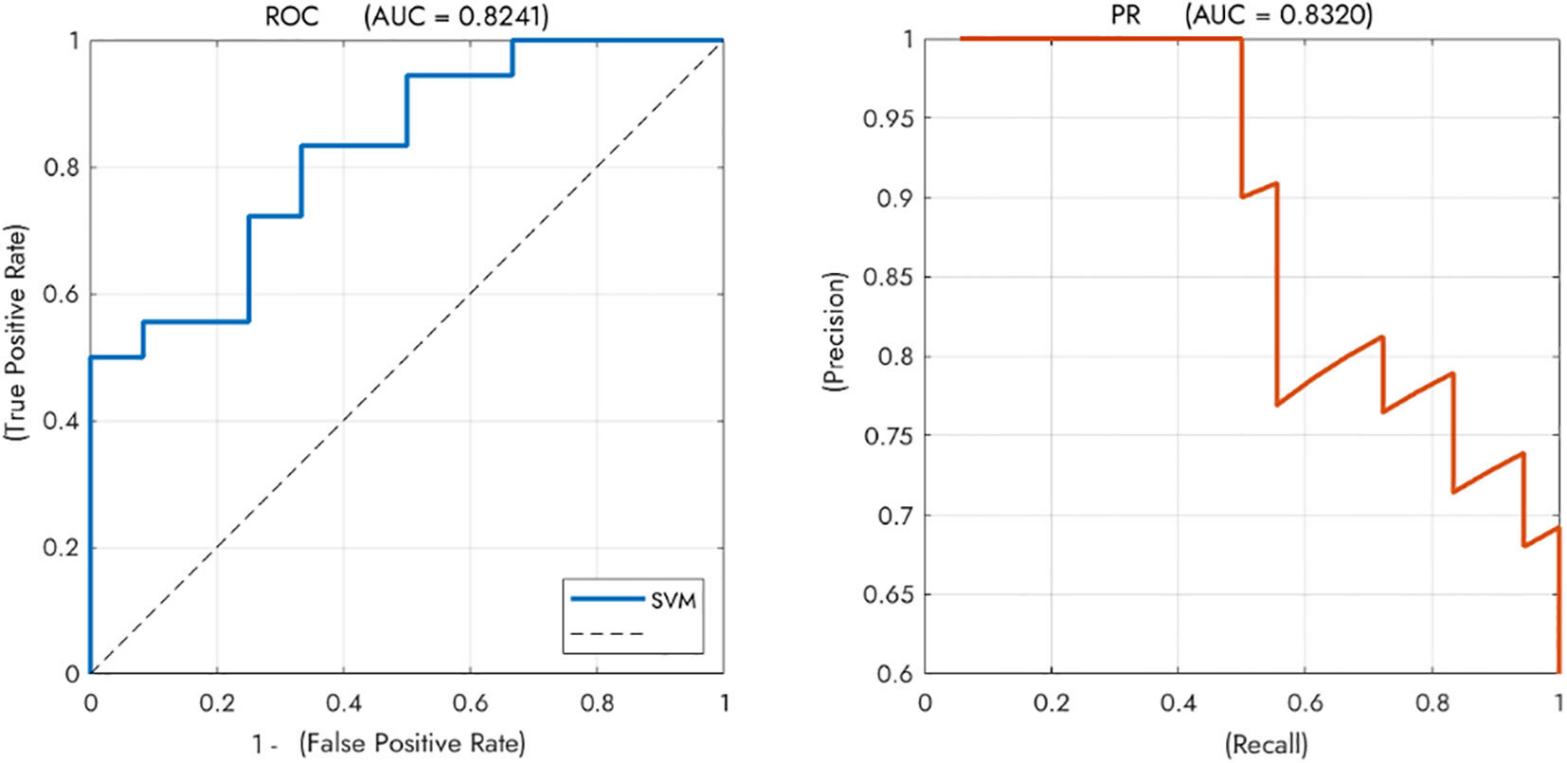
Performance of the SVM classifier for discriminating between GD and NGD patients. The classifier achieved good overall performance with an Area Under the ROC Curve (AUC) of 0.8241. The Precision-Recall curve (AUPRC = 0.8320) further supports the model’s effectiveness. *Note.* SVM, Support Vector Machine; NGD, Depression without gastrointestinal symptoms; GD, Gastrointestinal Depression. .

As shown in **Figure 5**, the SVM classifier demonstrated good performance in distinguishing GD from NGD patients on the independent test set, achieving an overall accuracy of 70.0% (21 of 30 patients correctly classified). The model’s discriminative ability was further supported by an Area Under the ROC Curve (AUC) of 0.824 and an Area Under the Precision-Recall Curve (AUPRC) of 0.832.

## 4 Discussion

By integrating a large-scale normative dataset with clinical fMRI data, this study systematically investigated the neural circuit basis of GI symptoms in patients with MDD. We revealed that aberrant FC between a specific insular subregion—termed the GD-pINS—and the GN, previously defined by gastric rhythm, is a key neural substrate for GI symptoms in MDD. Our findings identified a graded pattern of connectivity strength, where the NGD group exhibited the lowest connectivity, the GD group showed intermediate levels, and the HCs had the highest. Further pathway-specific analysis revealed that the differences between the GD and NGD groups were primarily driven by connections involving the Secondary Somatosensory Left (SIIl), where the GD group showed relatively higher FC. The strength of these specific connections was significantly and positively correlated with the severity of patients’ GI symptoms. Finally, a SVM model built upon these FC features demonstrated high accuracy in distinguishing between GD and NGD patients, highlighting the potential of this circuit as a biomarker for MDD subtyping.

A seemingly paradoxical finding of our study is the graded pattern of NGD < GD < HCs in the overall FC between the GD-pINS and the GN (**Figure 2B**). We propose that this phenomenon arises from the interplay of two opposing processes: a generalized reduction in connectivity common to MDD and a symptom-specific enhancement of connectivity related to GI symptoms. MDD is associated with reduced global brain connectivity (GBC), indicating a widespread decrease in the communication and coordination between different brain regions (55, 56). Specifically, the salience network, of which the insula is a critical component (57), shows decreased internal connectivity in depression (58, 59). Similarly, the sensorimotor network, which is closely associated with the posterior insula (60), also exhibits reduced intra-network connectivity in MDD (61, 62). Conversely, the insula also demonstrates heightened FC with other cortical regions in the context of somatic symptoms. Increased FC between the insula and other brain regions involved in pain processing and emotional regulation has been consistently reported in chronic pain conditions (63–66), including low back pain (65), chronic pelvic pain (67), and chronic pain after spinal cord injury (64), which lends credibility to our findings. The work by Avery et al. (2015) provides a compelling model, revealing a dissociation between task-evoked activity and resting-state FC in the insula (9). They noted that while insular activity might be blunted during interoceptive tasks, it maintains a pathological, hypervigilant mode of communication with emotional centers at rest (68). Synthesizing this evidence, we hypothesize that for GD patients, persistent GI symptoms act as a highly salient endogenous stimulus. One possible interpretation is that this persistent interoceptive signaling drives a maladaptive neural response in the specific gastro-related circuit (GD-pINS-GN), manifesting as a relative enhancement of connectivity. This occurs against a backdrop of globally reduced connectivity in both patient groups compared to HCs and serves to differentiate GD from NGD patients.

Our pathway-specific analysis further elucidates that the key differentiator between the GD and NGD subgroups is not a simple global shift in network connectivity, but rather the remodeling of specific information processing pathways. The results point unequivocally to aberrant connectivity between the GD-pINS and the SII (**Figures 3**, **4B**; **Table 2**). Located in the parietal operculum, the SII is a higher-order sensory integration hub (69). Unlike the primary somatosensory cortex (SI), which is mainly responsible for sensory localization and discrimination, the SII is involved in more complex processes, including bilateral sensory integration, tactile memory, and, crucially, visceral and pain signal processing (70). The SII is consistently activated during visceral stimulation (e.g., rectal distension) and is closely associated with the negative affective component (i.e., unpleasantness) of pain (71, 72). Somatosensory amplification, a core feature of somatoform disorders and highly comorbid with depression and anxiety (68), has been linked to altered connectivity within the sensorimotor and salience networks (73, 74). Collectively, we propose that the observed GD-pINS-SII hyperconnectivity may lead to somatosensory amplification, causing patients to subjectively experience normal gastric afferent signals as pain, bloating, or nausea. The significant positive correlation between this pathway’s connectivity strength and GDI scores provides direct support for this hypothesis. However, additional mechanisms may also underlie this observation. Chronic low-grade inflammation, a well-established correlate of depression (75), can alter interoceptive sensitivity and potentially modulate resting-state connectivity within this circuit (76, 77). Another possibility is that the increased connectivity reflects a top-down attentional bias in which individuals with GI distress display heightened vigilance toward gut-related sensations even at rest (78). Further studies are warranted to disentangle these contributing factors.

Another notable finding is the right-sided lateralization of abnormal connectivity, with more numerous and significant aberrant pathways observed between the right GD-pINS and the GN compared to the left. This observation aligns with extensive anatomical and functional evidence for the functional division of labor between the bilateral insulae (79). The right insula is tightly linked to sympathetic nervous system activity, negative emotions, and conscious awareness of internal bodily states (i.e., interoception) (65, 80). In contrast, the left insula is more associated with parasympathetic activity, positive emotions, and approach-related behaviors (81–84). Given this, the stronger and more numerous aberrant connections originating from the right GD-pINS likely reflect this functional lateralization.

The SVM classifier, capable of distinguishing between GD and NGD patients based on GD-pINS-GN connectivity features, provides proof-of-concept for the potential of fMRI-based FC features to serve as an objective biomarker. Furthermore, our findings provide a rationale for developing targeted neuromodulation therapies. The vagus nerve is the primary afferent pathway from the stomach (85), with its signals being integrated in the brainstem and insula (86, 87). Transcutaneous auricular vagus nerve stimulation (taVNS), a therapy that modulates this pathway, holds promise for normalizing this aberrant connectivity to alleviate GI symptoms (88).

Several limitations of this study should be acknowledged. First, the cross-sectional design precludes causal inferences. We cannot determine whether the aberrant FC is a cause or a consequence of the GI symptoms. Second, most patients were receiving psychotropic medication at the time of scanning. Although we found no significant group differences in medication rates or classes (**Table 1**), these agents may still have influenced resting-state FC (89, 90). Thus, medication effects may have contributed to the observed differences between MDD patients and healthy controls. These findings should therefore be interpreted with caution, and studies in drug-naïve, first-episode patients are needed to disentangle disorder-related changes from treatment effects. Third, while this study used a GN template defined by gastric rhythms, we did not acquire direct physiological measures of gastric activity (e.g., electrogastrogram) in our clinical cohort. Therefore, our findings relate fMRI connectivity to self-reported GI symptoms rather than to real-time physiological gastric states. Fourth, we did not assess participants’ dietary habits, which are known to significantly influence gastrointestinal function and symptoms (91, 92). Future studies investigating the neural correlates of GI distress in depression would benefit from incorporating assessments of dietary patterns. Fifth, our exploratory seed-to-seed analysis did not correct for multiple comparisons. While the identified pathways showed clustering, these findings should be considered preliminary. Sixth, our subgroup sample sizes (NGD n=42, GD n=58) were relatively modest, which may limit the statistical power of our subgroup comparisons and the stability of the classifier’s performance. Our machine learning analysis, while promising, does not guarantee generalizability. Future studies aiming to develop a clinically viable biomarker must validate these findings using more robust methods, such as evaluation on a larger, entirely independent dataset. Finally, our assessment of GI symptoms relied on the GDI, a composite score derived from three items of the PHQ-15. While the PHQ-15 is a well-validated tool for somatic symptoms and our approach provided a concise and specific index for GI-related distress, we acknowledge that it is not a comprehensive gastroenterological instrument. This brief measure does not capture the full spectrum, frequency, or chronicity of GI symptoms with the detail afforded by specialized scales (e.g., the Gastrointestinal Symptom Rating Scale). Future research should therefore aim to replicate these findings using more dedicated gastroenterological assessments to explore the neural correlates of more nuanced symptom profiles.

## 5 Conclusion

In conclusion, this study suggests that altered FC between the GD-pINS and the GN may represent a neural substrate for GI symptoms in MDD. Our results indicate that against a background of generally reduced connectivity in MDD, patients with GI symptoms exhibit a relative enhancement in specific pathways involving the SII. This altered connectivity, which correlates with symptom severity, might reflect a mechanism of somatosensory amplification. These findings advance the neurobiological framework of somatic depression, highlighting this circuit’s potential as a biomarker for subtyping and a therapeutic target.

## Data Availability

The raw data supporting the conclusions of this article will be made available by the authors, without undue reservation.
