# Supplementary material for: A neural signature for gastrointestinal symptoms in depression: insula-gastric connectivity predicts symptom severity
Source: Front Psychiatry. 2025 Nov 19;16:1672148. doi: 10.3389/fpsyt.2025.1672148 (PMC12673926; doi:10.3389/fpsyt.2025.1672148)
Supplement: Supplementary file 1 [file DataSheet1.pdf]

SUPPLEMENTARY INFORMATION

A Neural Signature for Gastrointestinal Symptoms in Depression: Insula-Gastric Connectivity Predicts Symptom Severity

Qi *et al.*

Contents	Page
**Supplementary Tables**	**2**
**Supplementary Table 1.** Composition of the Gastrointestinal Discomfort Index (GDI) Derived from Patient Health Questionnaire-15 (PHQ-15) Items.	2
**Supplementary Table 2.** Node Definitions for the Gastric Network.	3
**Supplementary References**	**9**

Supplementary Table 1. Composition of the Gastrointestinal Discomfort Index (GDI) Derived from Patient Health Questionnaire-15 (PHQ-15) Items.

"During the past 2 weeks, how much have you been bothered by any of the following problems?"	Not bothered at all	Bothered a little	Bothered a lot
a. Stomach pain	0	1	2
b. Constipation, loose bowels, or diarrhea	0	1	2
c. Nausea, gas, or indigestion	0	1	2

Note. The GDI was constructed based on three items from the PHQ-15 assessing gastrointestinal symptoms. Each item was scored on a 3-point scale: 0 ("Not bothered at all"), 1 ("Bothered a little"), and 2 ("Bothered a lot"). The GDI is the sum of these three scores, with a total possible range of 0 to 6.

Supplementary Table 2. Node Definitions for the Gastric Network.

Node name	Abbreviation	Brain Regions	Label AAL	Abbreviation	MNI of max t		
					X	Y	Z
Primary Somatosensory Right	SIr	Right Postcentral Gyrus	Postcentral R	PoCG-R	45	-28	46
Secondary Somatosensory Right	SIIr	Right Rolandic Operculum	Rolandic Oper R	ROL-R	54	-22	13
		Right Heschl's Gyrus	Heschl R	HES-R	51	-19	10
		Right Superior Temporal Gyrus	Temporal Sup R	STG-R	57	-25	13
Secondary Somatosensory Left	SIIl	Left Postcentral Gyrus	Postcentral L	PoCG-L	-66	-22	16
		Left Supramarginal	SupraMarginal L	SMG-L	-60	-25	16

		Gyrus					
		Left Heschl's Gyrus	Heschl L	HES-L	-54	-16	7
		Left Superior Temporal Gyrus	Temporal Sup L	STG-L	-54	-19	7
Medial Wall Motor Regions	MWM	Left Supplementary Motor Area	Supp Motor Area L	SMA-L	0	-13	49
		RightSupplementary Motor Area	Supp Motor Area R	SMA-R	9	4	52
		Left Middle Cingulate Gyrus	Cingulum Mid L	MCG-L	0	-22	43
		Right Middle Cingulate Gyrus	Cingulum Mid R	MCG-R	6	-10	43
Posterior Cingulate Sulcus	pCS	Left Middle	Cingulum Mid L	MCG-L	-9	-37	52

		Cingulate Gyrus					
		Right Middle Cingulate Gyrus	Cingulum Mid R	MCG-R	6	-37	49
		Left Precuneus	Precuneus L	PCUN-L	0	-37	55
		Right Precuneus	Precuneus R	PCUN-R	6	-40	52
		Right Paracentral Lobule	Paracentral Lobule R	PCL-R	15	-40	55
Dorsal Precuneus	dPrec	Left Precuneus	Precuneus L	PCUN-L	-3	-67	61
		Right Precuneus	Precuneus R	PCUN-R	3	-64	64
Dorsal Precuneus Left Anterior	ladPrec	Left Precuneus	Precuneus L	PCUN-L	-6	-55	73
		Left Paracentral Lobule	Paracentral Lobule L	PCL-L	-6	-34	76
Occipital Ventral	vOcc	Left Calcarine Sulcus	Calcarine L	CAL-L	0	-64	10

		Left Lingual Gyrus	Lingual L	LING-L	0	-67	7
		Right Lingual Gyrus	Lingual R	LING-R	6	-70	-5
		Right Cerebellum Lobe VI	Cerebellum 6 R	CER6-R	12	-70	-14
		Vermis Lobules IV-V	Vermis 4 5	VER4/5	0	-61	-2
		Vermis Lobule VI	Vermis 6	VER6	3	-70	-8
Occipital Dorsal	dOcc	Left Calcarine Sulcus	Calcarine L	CAL-L	3	-76	16
		Left Cuneus	Cuneus L	CUN-L	-3	-85	31
		Right Cuneus	Cuneus R	CUN-R	6	-76	28
Extrastriate Body Area Right	EBA	Right Inferior Occipital Gyrus	Occipital Inf R	IOG-R	48	-76	-14

		Right Inferior Temporal Gyrus	Temporal Inf R	ITG-R	57	-70	-11
		Right Cerebellum Crus I	Cerebellum Crus1 R	CERC1-R	45	-82	-26
Superior Parieto-Occipital Sulcus	sPOS	Right Cuneus	Cuneus R	CUN-R	21	-76	43
		Superior Occipital Gyrus Right	Occipital Sup R	SOG-R	24	-79	43
Retrosplenial Cortex	RSC	Left Posterior Cingulate Gyrus	Cingulum Post L	PCG-L	-6	-43	10
		Right Posterior Cingulate Gyrus	Cingulum Post R	PCG-R	6	-40	7
		Right Lingual Gyrus	Lingual R	LING-R	9	-37	-2
		Left Precuneus	Precuneus L	PCUN-L	-6	-49	7

Note. The table details the 12 nodes and 39 constituent brain regions of the gastric network used in this study. This network was originally defined and reported by Rebollo et al. (2018) (1). For each brain region, the table lists the corresponding AAL atlas label and the MNI coordinates of the peak voxel. AAL, Automated Anatomical Labeling; MNI, Montreal Neurological Institute; L, Left; R, Right.

Supplementary References

1. Rebollo I, Devauchelle A-D, Béranger B, Tallon-Baudry C. Stomach-brain synchrony reveals a novel, delayed-connectivity resting-state network in humans. *Elife* (2018) 7:e33321. doi: 10.7554/eLife.33321
